# Overexpressing the HD-Zip class II transcription factor *EcHB1* from *Eucalyptus camaldulensis* increased the leaf photosynthesis and drought tolerance of *Eucalyptus*

**DOI:** 10.1038/s41598-019-50610-5

**Published:** 2019-10-01

**Authors:** Keisuke Sasaki, Yuuki Ida, Sakihito Kitajima, Tetsu Kawazu, Takashi Hibino, Yuko T. Hanba

**Affiliations:** 1 Faculty of Applied Biology, Kyoto Institute of Technology, Matsugasaki, Sakyo-ku, Kyoto 606-8585 Japan; 2 Forestry Research Institute, Oji Holdings Corporation, 24-9 Nobono-cho, Kameyama, Mie 519-0212 Japan; 3 Present Address: K-Plantech, 3085-15 Kobe, Tsu, Mie 514-0065 Japan; 4 Present Address: Pine Chemicals Development, R&D Center, R&D Company, HARIMA CHEMICALS INC., 5-9-3 Tokodai, Tsukuba, Ibaraki 300-2635 Japan

**Keywords:** Photosynthesis, Plant stress responses

## Abstract

Alteration in the leaf mesophyll anatomy by genetic modification is potentially a promising tool for improving the physiological functions of trees by improving leaf photosynthesis. Homeodomain leucine zipper (HD-Zip) transcription factors are candidates for anatomical alterations of leaves through modification of cell multiplication, differentiation, and expansion. Full-length cDNA encoding a *Eucalyptus camaldulensis* HD-Zip class II transcription factor (EcHB1) was over-expressed *in vivo* in the hybrid *Eucalyptus* GUT5 generated from *Eucalyptus grandis* and *Eucalyptus urophylla*. Overexpression of *EcHB1* induced significant modification in the mesophyll anatomy of *Eucalyptus* with enhancements in the number of cells and chloroplasts on a leaf-area basis. The leaf-area-based photosynthesis of *Eucalyptus* was improved in the *EcHB1*-overexpression lines, which was due to both enhanced CO_2_ diffusion into chloroplasts and increased photosynthetic biochemical functions through increased number of chloroplasts per unit leaf area. Additionally, overexpression of *EcHB1* suppressed defoliation and thus improved the growth of *Eucalyptus* trees under drought stress, which was a result of reduced water loss from trees due to the reduction in leaf area with no changes in stomatal morphology. These results gave us new insights into the role of the HD-Zip II gene.

## Introduction

Leaf photosynthetic functions are key traits for the determination of tree growth. Enhancement of leaf photosynthesis will thus be a possible measure for improvement in the productivity of forest trees. *Eucalyptus* species are one of the most widely-planted multipurpose tree species in the world^1,2^, which are used as a source of paper pulp, wood, and timber. *Eucalyptus* species have a high growth rate and high photosynthetic rate, while soil water availability strongly limits their growth^3,4^; therefore, improvement in leaf photosynthesis and tree growth, as well as raising drought tolerance under limited water resources, are important issues for improving the production of *Eucalyptus* trees.

One of the promising measures to improve leaf photosynthesis is to modify the leaf mesophyll anatomy. Leaf photosynthesis is limited by (1) CO_2_ diffusion through the stomata, (2) CO_2_ diffusion through the mesophyll, and (3) photosynthetic biochemistry, in which both CO_2_ diffusion through the mesophyll and photosynthetic biochemistry are strongly affected by the leaf mesophyll anatomy, in particular the number of chloroplasts^5–8^. Thick and dense leaf mesophyll tissue generally has a high number of chloroplasts and thus involves high photosynthetic functions through high mesophyll CO_2_ diffusion and a large amount of carboxylation enzymes within a single species^9–11^ and among different species of angiosperms and ferns^8^. Furthermore, the above alterations in the leaf mesophyll anatomy also potentially improve both the growth and drought tolerance of tree leaves through improvement in the water-use efficiency, when an increase in water-use efficiency is not accompanied by a concurrent decline in CO_2_ fixation limited by mesophyll or photosynthetic biochemistry^12^. Although a high photosynthetic rate generally corresponds to high water loss from the stomata due to large stomatal conductance and high hydraulic conductance among different species^13^, an increase in the photosynthetic rate with little change in stomatal conductance has been reported in *Eucalyptus* leaves overexpressing radish aquaporin *RsPIP2;1*^14^.

In this study, we focused on *EcHB1* from *Eucalyptus* as a candidate for improving tree photosynthesis, growth, and drought tolerance through changes in the leaf anatomy. *EcHB1* is a *Eucalyptus camaldulensis* homolog of the homeobox protein HAT22 of *Arabidopsis thaliana* (AT4G37790). They belong to the HD-Zip II transcription factor family which contains a homeodomain and leucine zipper motif. *EcHB1* was originally isolated as a factor that controls the expression of multiple genes encoding enzymes for cell wall biosynthesis pathways^15^. In our previous study^15^, we generated *Eucalyptus* plants overexpressing *EcHB1* under the control of a CaMV 35 S promoter, and reported an increase in the plant growth and alterations in flower morphology that suggest functional roles of *EcHB1* on the cell growth in multiple plant organs including the leaves. In fact, we obtained significant alterations in leaf morphology in *Eucalyptus* trees overexpressing *EcHB1* in our preliminary experiment. HD-Zip transcription factors regulate plant growth responses through suppression or promotion of cell multiplication, differentiation, and expansion through phytohormone-regulated networks^16,17^. Previous genetic modification efforts for improvement in the growth and production of trees have focused on cellulose modification, imparting tolerance to biotic and abiotic stresses and modification of lignin content^18^, in which a decrease in the lignin content through the suppression of some genes, including Pt4CL1 encoding 4-coumarate:coenzyme A ligase (*4CL*), cinnamyl alcohol dehydrogenase (CAD), and caffeate/5-hydroxyferulate O-methyltransferase (COMT), has been studied^19–21^. However, adequate lignification is essential for mechanical support and water transport in trees; the down-regulation of two genes encoding two *4CL*s causes increased xylem vulnerability to embolism and decreased tree growth of poplar^22^.

The aims of the present study are to test the hypothesis that overexpression of *EcHB1*, an HD-Zip II transcription factor from *Eucalyptus camaldulensis*, improves the leaf photosynthetic function, growth, and drought tolerance through alterations in the mesophyll anatomy in *Eucalyptus* trees. Alterations in the following three factors: CO_2_ diffusion through the stomata, CO_2_ diffusion through the mesophyll, and photosynthetic biochemistry will be discussed as possible mechanisms explaining changes in leaf photosynthetic functions. Additionally, the number of leaves and growth in response to soil drought were examined for assessing alteration in the drought tolerance of *Eucalyptus* trees through overexpression of *EcHB1*.

## Results

### Gene expression levels and phenotypes

**Figure 1:**
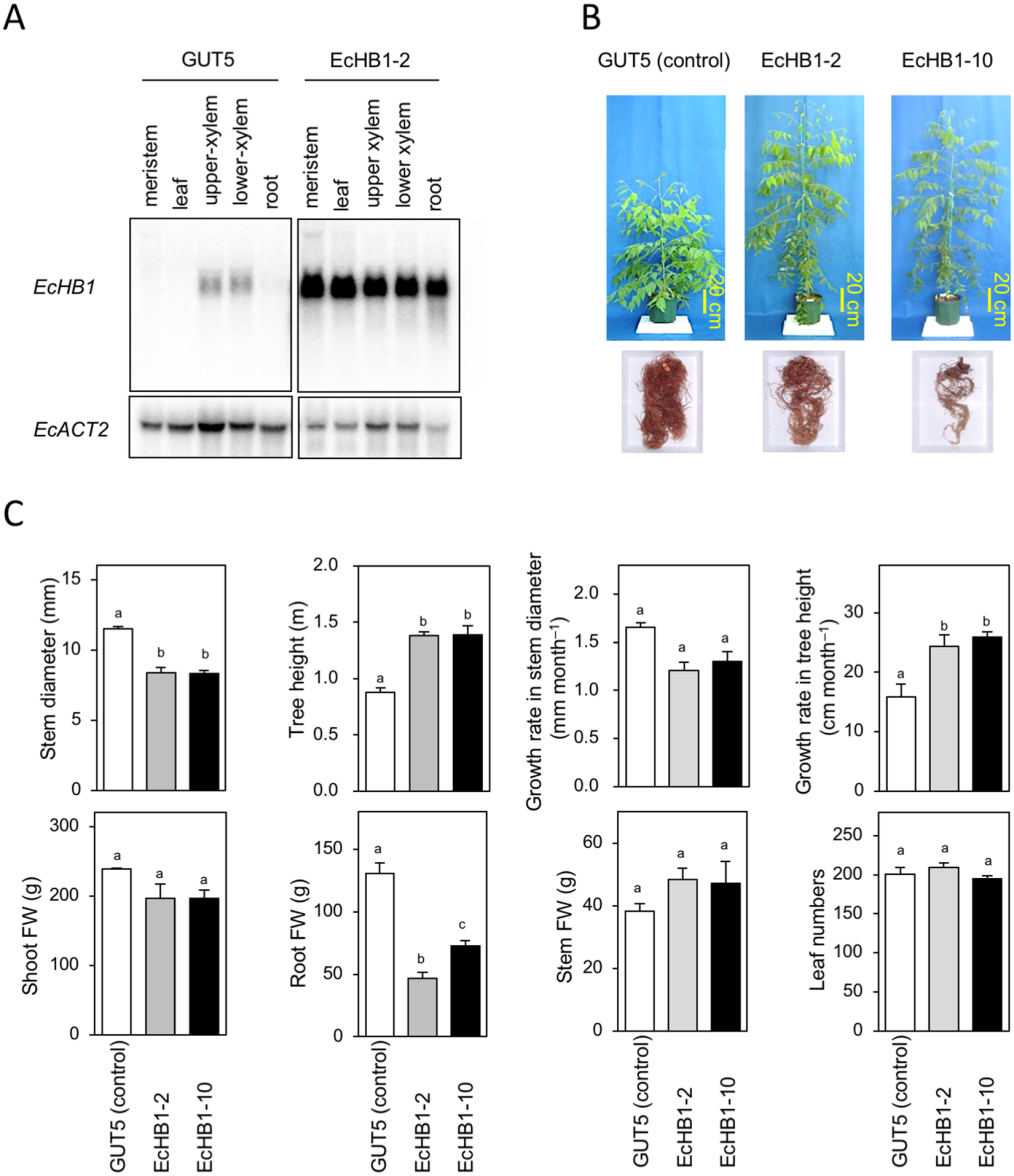
(**A**) Gene expression levels measured using Northern blot analysis of the meristem, leaf, upper xylem, lower xylem, and root of the control GUT5 and EcHB1-overexpressed line EcHB1-2, in which actin2 of *Eucalyptus camaldulensis* (EcACT2) was used as the control (see Supplemental Information). (**B**) Phenotypes of the control (GUT5) and *EcHB1*-overexpressed lines (EcHB1-2 and EcHB1-10), grown for 12 months. (**C**) Tree growth traits such as stem diameter, tree height, growth rate in stem diameter and tree height, shoot fresh weight (FW), root FW and leaf number. Blank, gray, and filled bars indicate GUT5, EcHB1-2, and EcHB1-10, respectively. Means with standard errors are shown for 3–12 trees. Differences between lines were analyzed using one-way ANOVA, in which different letters indicate that the difference was statistically significant (*P* < 0.05).

RT-PCR analysis showed that in *Eucalyptus* leaves, expression levels of *EcHB1* in the overexpressed lines EcHB1-2 and EcHB1-10 were much higher than those in the control line GUT5 (14-23-fold, Fig. S1A). Northern blot analysis revealed that in the transgenic line EcHB1-2, *EcHB1* was highly expressed in the meristem, leaf, upper xylem, lower xylem, and roots, while *EcHB1* expression levels in these tree tissues were very low or almost negligible in the control line GUT5 (Fig. 1A).

Alterations in some of the tree phenotypes were obtained for the two transgenic *Eucalyptus* lines EcHB1-2 and EcHB1-10 compared to the control line GUT5 (Fig. 1B). In the overexpressed lines, tree heights and their growth rate were 1.5-1.6-fold, while diameters at the base of the stems were 0.7-fold and the root fresh weights were 0.4-0.6-fold those of the GUT5 (Fig. 1C). The shoot and stem fresh weights, stem growth rate, and leaf number were not changed in the overexpressed lines.

### Leaf and photosynthetic traits

**Figure 2:**
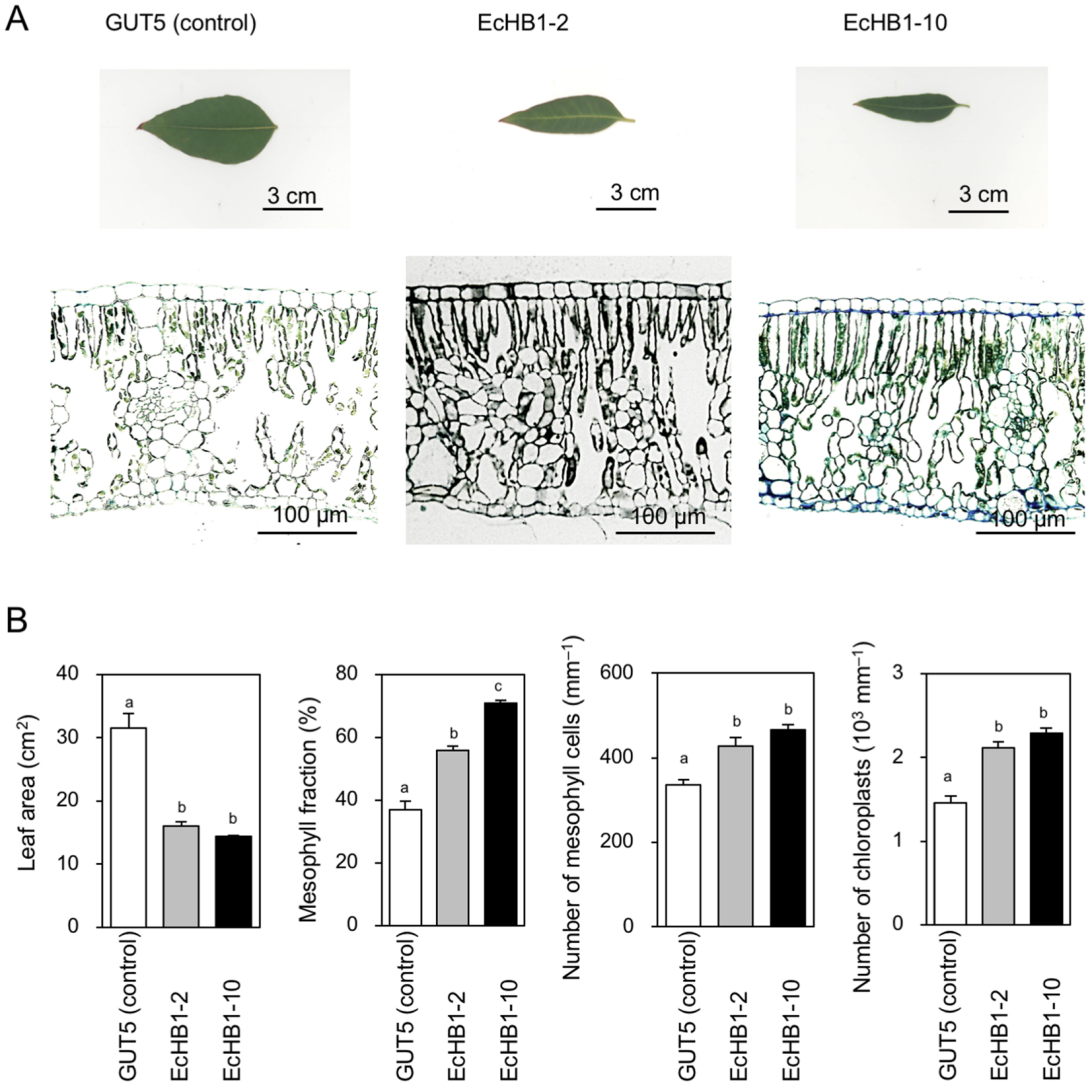
(**A**) Images of the leaves of *Eucalyptus* and their transverse sections. (**B**) Leaf area, leaf mesophyll fraction, number of mesophyll cells and chloroplasts, in which means with standard errors are shown for 5–18 fully-expanded mature leaves. Blank, gray, and filled bars indicate GUT5, EcHB1-2, and EcHB1-10, respectively. Differences between lines were analyzed using one-way ANOVA, in which different letters indicate that the difference was statistically significant (*P* < 0.05).

Significant alterations were again observed in the leaf morphological and anatomical traits in the transgenic lines EcHB1-2 and EcHB1-10 (Fig. 2A). These transgenic lines had densely-packed small leaves with increased number of chloroplasts; in these transgenic lines, the leaf area was 0.5-fold, while the mesophyll fractions (ratio of mesophyll tissues in leaf section), number of mesophyll cells, and chloroplasts was 1.3-1.9-fold of the control line GUT5 (Fig. 2B).

**Figure 3:**
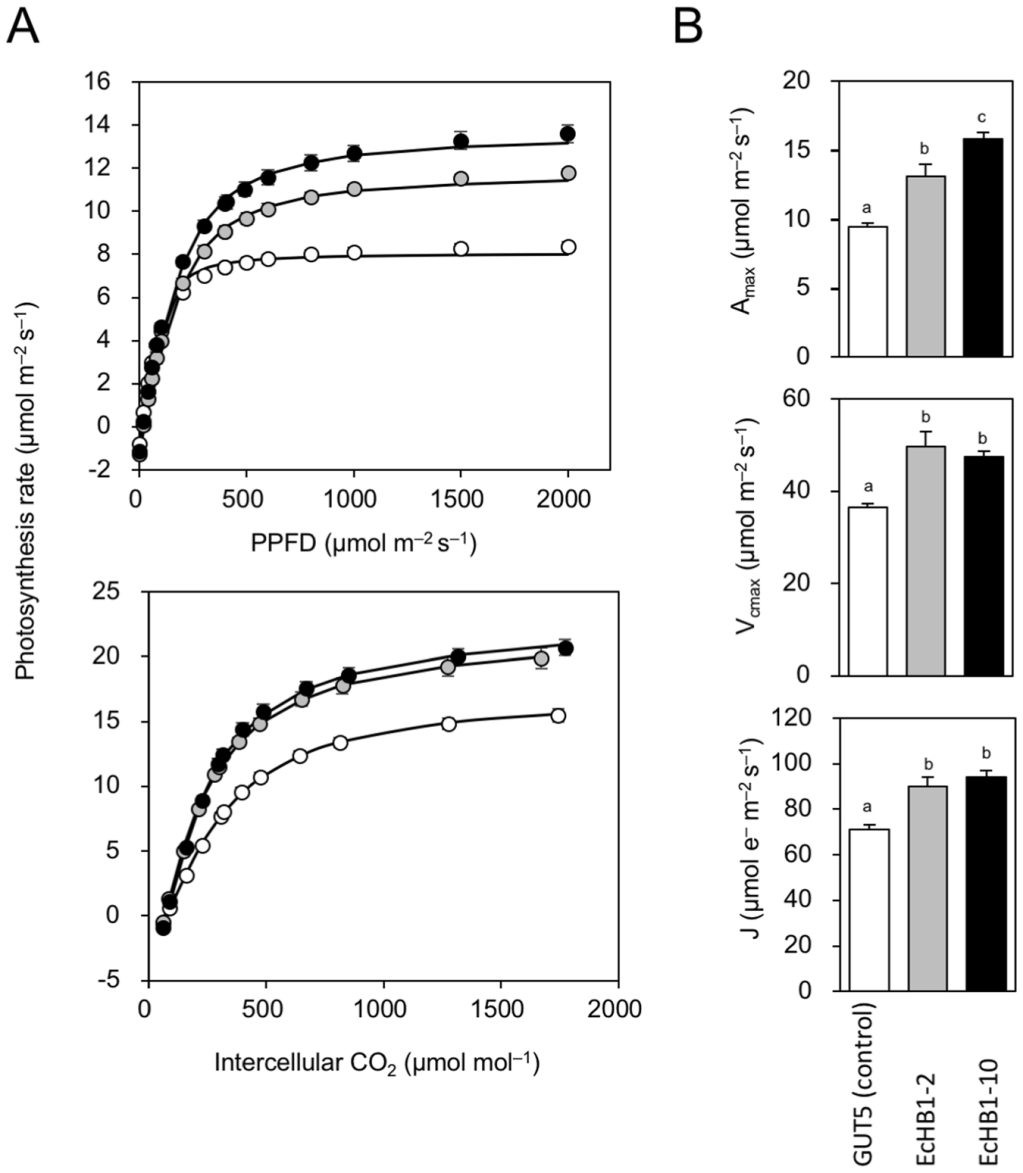
(**A**) Photosynthetic light response curves and *A*-*C*_i_ curves in the leaves of *Eucalyptus*. (**B**) Photosynthetic parameters such as maximum photosynthetic rate (*A*_max_), maximum carboxylation rate (*V*_cmax_), and maximum electron transport rate (*J*) estimated from light response curves and *A*-*C*_i_ curves in the leaves of *Eucalyptus*. Means with standard errors are shown for 4–12 fully-expanded mature leaves. Blank, gray, and filled bars or symbols indicate GUT5, EcHB1-2, and EcHB1-10, respectively. Differences between lines were analyzed using one-way ANOVA, in which different letters indicate that the difference was statistically significant (*P* < 0.05).

As a result of the dense mesophyll tissues and increased number of chloroplasts, the transgenic lines EcHB1-2 and EcHB1-10 had a high area-based photosynthetic capacity compared to the control line GUT5 (Fig. 3A). The maximum photosynthetic rate (*A*_max_), maximum Rubisco carboxylation rate (*V*_cmax_), and photosynthetic electron transport rate (*J*) in the transgenic lines EcHB1-2 and EcHB1-10 were 1.3-1.7-fold those of the control line GUT5 (Fig. 3B).

**Figure 4:**
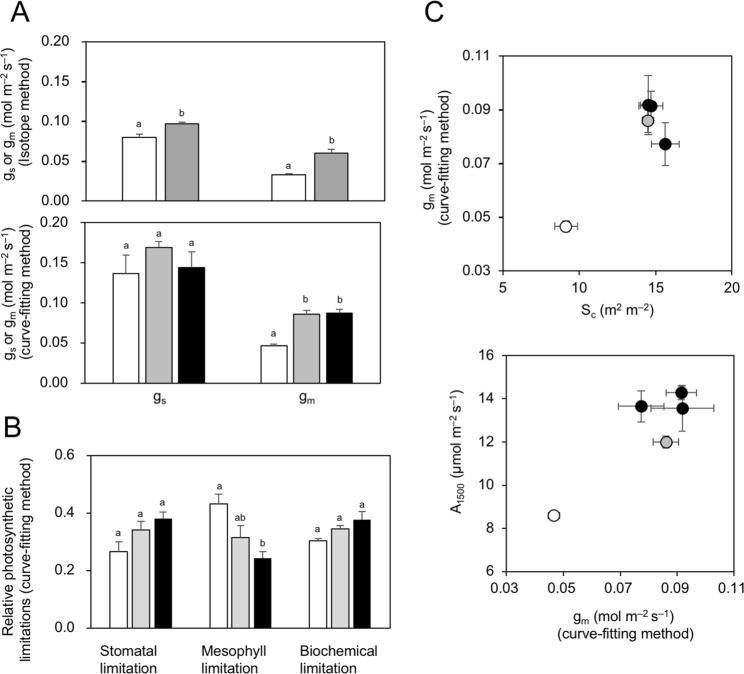
(**A**) Stomatal conductance (*g*_s_) or mesophyll conductance (*g*_m_) estimated using the isotope method or the curve-fitting method in the leaves of *Eucalyptus*. The data for EcHB1-10 obtained by the isotope method were eliminated because of the unexpected effect of senescence. (**B**) Photosynthetic limitations of the stomata, mesophyll, or biochemical traits. The differences between the control and *EcHB1*-overexpressed lines were tested using using the *t*-test (isotope method) or one-way ANOVA (curve-fitting method), with different letters indicating a statistically significant difference (p < 0.05). Means with standard errors are shown for 4–12 fully-expanded mature leaves. (**C**) Relationship between the surface area of chloroplasts facing intercellular airspaces (*S*_c_) and *g*_m_, and between *g*_m_ and the photosynthesis rate at 1500 μmol m^−2^ s^−1^ of PPFD (*A*_1500_). Means with standard errors for individual trees are plotted. Blank, gray, and filled bars or symbols indicate GUT5, EcHB1-2, and EcHB1-10, respectively.

We determined the CO_2_ diffusional limitations during photosynthesis in the *Eucalyptus* leaves (Fig. 4). Stomatal conductance (*g*_s_) in the transgenic lines was slightly higher (1.2-fold) or similar to that in the control lines, while mesophyll conductance (*g*_m_) in the transgenic lines EcHB1-2 and EcHB1-10 was 1.8-1.9-fold that of the control line GUT5 (Fig. 4A). Although the stomatal and biochemical limitations were similar between the lines, the mesophyll limitations of leaf photosynthesis were smaller in the EcHB1-10 line compared to the control line GUT5 (Fig. 4B). The higher *g*_m_ in the transgenic lines EcHB1-2 and EcHB1-10 correlated to a higher chloroplast surface area exposed to intercellular airspaces (*S*_c_), and the higher photosynthetic rate at 1500 µmol m^−2^ s^−1^ of PPFD (*A*_1500_) in the transgenic lines correlated to a higher *g*_m_ (Fig. 4C).

The data for photosynthesis and leaf morphological or anatomical traits were pooled for the two transgenic lines that had high *EcHB1* expression levels (EcHB1-2 and EcHB1-10), and then compared to those of the control line GUT5 (Table 1). Leaf photosynthetic traits were increased in *EcHB1*-overexpressed lines except for stomatal conductance (*g*_s_), which corresponded to an increase in the anatomical factors enhancing CO_2_ diffusion (*S*_c_, 1.6-fold) with no change in the cell wall thickness, which would inhibit CO_2_ diffusion. The increase in the number of mesophyll cells (1.4-fold) was accompanied by decreases in the palisade and epidermal cell width (0.8-fold), and an increase in the number of chloroplasts (1.5-fold) was followed by decreases in the width and thickness of the chloroplasts (0.8–0.9-fold). A higher leaf mass per area (LMA, 1.2-fold) while a similar mesophyll thickness was obtained. Stomatal traits including the stomatal size and density were similar between the *EcHB1*-overexpressed lines and GUT5.

**Table 1:** Mean values of photosynthetic, anatomical, and morphological traits of the 4–22 fully-expanded mature leaves in the 4-year-old trees.

Trait	GUT5(control)	EcHB1-2 + EcHB1-10 (overexpression)
**Leaf photosynthetic traits**		
*A*_max_ (μmol m^−2^ s^−1^)	9.4 (0.3)	14.8 (0.6)^***^
*g*_s_ (mol m^−2^ s^−1^)	0.0796 (0.0063)	0.0966 (0.0034)^n.s.^
*g*_m_ (mol m^−2^ s^−1^)	0.0325 (0.0022)	0.0598 (0.0075)^***^
*A*_1500_/*E*_1500_ (μmol CO_2_ mmol H_2_O^−1^)	3.01 (0.13)	3.96 (0.12)^***^
*V*_cmax_ (μmol m^−2^ s^−1^)	36.6 (0.7)	45.5 (1.5)^***^
*J* (μmol electrons m^−2^ s^−1^)	71.1 (2.2)	89.5 (3)^***^
**Leaf anatomical traits related to mesophyll CO**_**2**_ **diffusion**		
*S*_c_ (m^2^ m^−2^)	9.1 (0.7)	14.7 (0.3)^***^
Mesophyll cell wall thickness (μm)	0.183 (0.014)	0.208 (0.005)^n.s.^
**Number of cells and size**		
Mesophyll cell number (mm^−1^ section)	336 (14)	457 (11)^***^
Mesophyll palisade cell width (μm)	9.7 (0.4)	7.8 (0.2)^***^
Mesophyll palisade cell length (μm)	64.3 (1.8)	63.6 (1.7)^n.s.^
Mesophyll spongy cell width (μm)	8.9 (0.3)	9.4 (0.3)^n.s.^
Mesophyll spongy cell length (μm)	19.9 (1.4)	26.5 (0.5)^***^
Epidermal cell width (μm)	22.6 (1.6)	18.2 (0.5)^*^
Epidermal cell thickness (μm)	18.5 (0.5)	15.9 (0.2)^***^
**Number of chloroplasts and size**		
Chloroplasts (10^3^ mm^−1^ section)	1.46 (0.08)	2.24 (0.06)^***^
Chloroplast width (μm)	5.23 (0.15)	4.02 (0.11)^***^
Chloroplast thickness (μm)	1.26 (0.06)	1.09 (0.02)^**^
**Other leaf traits**		
Leaf area (cm^2^)	31.5 (2.3)	14.9 (0.3)^***^
Leaf mass per area (g m^−2^)	73.6 (3.5)	90.5 (3.8)^**^
Mesophyll thickness (μm)	256 (7)	247 (4)^n.s.^
Chlorophyll content (SPAD)	34.4 (0.8)	41.0 (0.6)^***^
**Stomatal morphological traits**		
Stomatal length (μm)	11.9 (0.3)	12.3 (0.3)^n.s.^
Stomatal width (μm)	6.26 (0.19)	6.17 (0.15)^n.s.^
Stomatal density (mm^−2^)	521 (44)	495 (39)^n.s.^

Means with standard errors in parenthesis are shown. Data obtained using the isotope method are presented for mesophyll conductance (*g*_m_). Anatomical data were obtained using light or electron micrographs (*n* = 25–120). Significant levels were tested by Welch’s t-test with differences shown as ^*^P < 0.05, ^**^P < 0.01 and ^***^P < 0.001. n.s. means no significant difference (*P* > 0.09).

**Figure 5:**
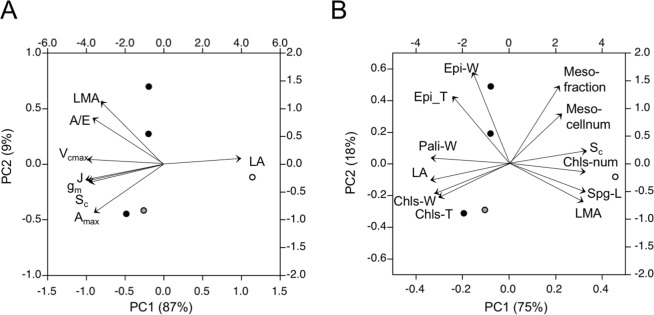
Biplot of the principle component analyses for leaf and photosynthetic traits where the differences between the control and *EcHB1*-overexpressed lines were statistically significant (see Table 1). Mean values of traits for individual trees were used for the analysis (*n* = 5), in which open, gray, and filled circles indicate the principle component scores for GUT5, EcHB1-2, and EcHB1-10, respectively. Arrows indicate factor loadings of each trait. (**a**) Leaf traits and photosynthetic parameters such as leaf area (LA), leaf mass per leaf area (LMA), surface area of chloroplasts facing intercellular airspaces (*S*_c_), maximum photosynthetic rate (*A*_max_), maximum carboxylation rate (*V*_cmax_), maximum electron transport rate (*J*), and transpiration efficiency (*A*/*E*). (**b**) Principle component analysis for the anatomical or morphological traits such as epidermal cell width and thickness (Epi-W, and Epi-T), palisade cell width (Pali-W), chloroplast width and thickness (Chls-W and Chls-T), LA, spongy cell length (Spg-L), number chloroplasts (Chls-num), mesophyll fraction and number of cells (Meso-fraction and Meso-cellnum), LMA, and *S*_c_.

Principle component analyses were performed for the traits that were different between the *EcHB1*-overexpressed lines and GUT5 (Table 1), among the traits that were related to leaf photosynthetic functions (Fig. 5A), and among the leaf anatomical traits (Fig. 5B). The first main axis (PC1) is considered to be suitable for explaining variabilities in the data for both analyses, because PC1 accounted for 75–87% of the total variability in the data. Gas exchange parameters, such as *A*_max_, *g*_m_, *V*_cmax_, *J*, and *A*/*E*, had negative loadings, and the anatomical traits of *S*_c_ and LMA also had negative loadings, while the leaf area (LA) had a positive loading over PC1 (Fig. 5A). Overall, the size of the cells or chloroplasts had negative loadings, which corresponded to the negative loading of LA over PC1 (Fig. 5B). In contrast, the number of cells, number of chloroplasts, and fraction of mesophyll cells had positive loadings, with positive loadings of LMA and *S*_c_. In these biplots, GUT5 plants had positive scores, while EcHB1-2 and EcHB1-10 had negative scores over PC1.

### Photosynthetic traits on a tree-basis and drought tolerance

The evaporation rate measured at 1500 μmol m^−2^ s^−1^ of PPFD (*E*_1500_) was smaller in the line EcHB1-2 than GUT on a tree-basis (0.6-fold) and on a leaf-basis (0.5-fold), while the line EcHB1-2 had a similar *E*_1500_ on a leaf-area basis to those in the line GUT5 (Table 2). On a tree-basis, photosynthesis measured at 1500 μmol m^−2^ s^−1^ of PPFD (*A*_1500_) and the surface area of the chloroplasts facing intercellular airspaces (*S*_c_) were similar between the line GUT5 and EcHB1-2, although in the transgenic line EcHB1-2, these traits were higher and lower on a leaf-area-basis and a whole-leaf-basis, respectively.

**Table 2:** Photosynthesis rate and evaporation rate measured at 1500 μmol m^−2^ s^−1^ of PPFD (*A*_1500_, *E*_1500_) and surface area of chloroplasts facing intercellular airspaces (*S*_c_) on a leaf-area basis, whole-leaf basis, and tree basis.

Parameter	Genotype	
	GUT5 (control)	EcHB1-2 (transgenic)
**Tree basis**		
*A*_1500_ (μmol s^−1^)	3.15 (0.24)	2.70 (0.19)^n.s.^
*E*_1500_ (mmol s^−1^)	1.11 (0.08)	0.68 (0.05)^***^
*S*_c_ (m^2^)	3.35 (0.25)	3.26 (0.23)^n.s.^
**Whole-leaf basis**		
Leaf area × *A*_1500_ (μmol s^−1^)	0.0271 (0.002)	0.0192 (0.008)^**^
Leaf area × *E*_1500_ (μmol s^−1^)	9.54 (0.69)	4.87 (0.2)^***^
Leaf area × *S*_c_ (10^−3^ m^2^)	28.8 (2.1)	23.2 (0.9)^*^
**Leaf-area basis**		
*A*_1500_ (μmol m^−2^ s^−1^)	8.59 (0.21)	12.0 (0.28)^***^
*E*_1500_ (mmol m^−2^ s^−1^)	3.03 (0.19)	3.04 (0.15)^n.s.^
*S*_c_ (m^2^ m^−2^)	9.1 (0.7)	14.5 (0.4)^***^

Mean values with standard errors in parenthesis are shown. Fully-expanded mature leaves (*n* = 4–6) and 8-month-old trees (*n* = 15–16) were used for the analysis. Significant levels of differences between GUT5 and EcHB1-2 were tested by Welch’s t-test with ^*^P < 0.05, ^**^P < 0.01 and ^***^P < 0.001. n.s. means no significant difference (*P* > 0.1).

**Figure 6:**
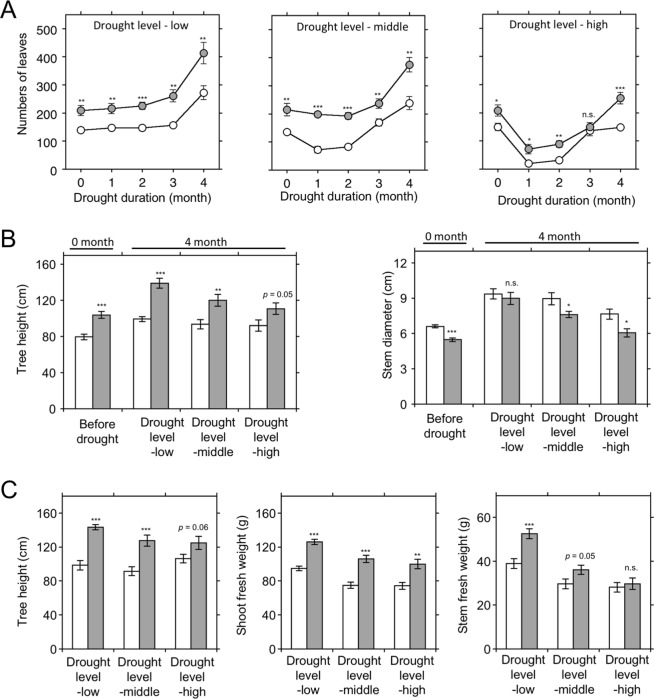
Growth response of the 8-month-old *Eucalyptus* trees to drought and the following re-watering treatment for the control (GUT5) and *EcHB1*-overexpressed line (EcHB1-2). Blank and gray symbols or bars indicate GUT5 and EcHB1-2, respectively. (**A**) Number of leaves during the 4-month drought experiment. (**B**) Tree height and stem diameter before and after the 4-month drought treatment. (**C**) Tree height and shoot and stem fresh weight after 3 months of re-watering. Means with standard errors are shown for 7–25 trees. The differences between GUT5 and EcHB1-2 were tested using the t-test, with asterisks indicating a significant difference with  ^*^P < 0.05, ^*^^*^P < 0.01, ^*^^*^^*^P < 0.001 and n.s. indicating the difference was not significant. *P*-values are shown when 0.05 < *P* < 0.1.

Overall, the drought and following recovery experiment showed enhanced drought tolerance in EcHB1-2 (Fig. 6). The number of leaves was higher in EcHB1-2 than that in GUT5 at all three drought levels during the 4-month drought experiment, except for the 3-month high-drought level (Fig. 6A). The tree height was higher in EcHB1-2 than that in GUT5 at all three drought levels after a 4-month drought (Fig. 6B). Stem diameter was similar in EcHB1-2 and GUT5 at the low-drought level, and differences between EcHB1-2 and GUT5 at the middle- and high-drought levels became less distinct compared to before the drought experiment. After the 3-month recovery experiment, enhanced growth was observed overall in the transgenic line EcHB1-2, in which differences between the lines were more significant under the low-drought level (Fig. 6C). The ratios of tree height, shoot fresh weights and stem fresh weights of EcHB1-2 to those of GUT5 were 1.4-1.5-fold, 1.3-1.4-fold, and 1.2-1.4-fold at the low-, middle-, and high-drought levels, respectively.

## Discussion

Significant enhancements in the area-based leaf photosynthetic functions were obtained in *Eucalyptus* trees overexpressing *EcHB1* (Fig. 3). This is the first study showing the effect of an HD-Zip class II transcription factor on the leaf photosynthetic function. Leaf photosynthesis is limited by 1) biochemical activities and 2) CO_2_ diffusion through the stomata and mesophyll^6–8^. Overexpression of *EcHB1* in the leaf induced significant alterations in the leaf mesophyll anatomy (Fig. 2, Table 1), which contributed to significant enhancements in the area-based leaf photosynthetic functions (Fig. 3) through enhancements in both the biochemical activities (*V*_cmax_ and *J*, Fig. 3) and CO_2_ diffusion through the mesophyll (*g*_m_, Fig. 4). The principle component analysis of leaf anatomical traits indicated that the marked increase in the mesophyll fraction (1.5-1.9-fold, Fig. 2B) was mainly due to the increase in the number of mesophyll cells (1.4-fold, Fig. 5B, Table 1). The increase in the number of mesophyll cells is related to an increase in the number of chloroplasts (1.5-fold, Fig. 5B, Table 1), which may induce enhancements in the photosynthetic biochemistry, e.g., the carboxylation enzyme Rubisco that regulates the velocity of carboxylation (*V*_cmax_) and photochemical efficiency and the Calvin cycle that regulates the electron transport rate (*J*, Fig. 3B). When the relationship between the leaf anatomical traits and biochemical parameters was analyzed, a high *V*_cmax_ corresponded to a large number of chloroplasts in angiosperms and ferns^8,23^.

Overexpression of *EcHB1* induced marked enhancement in the mesophyll diffusional conductance, *g*_m_ (1.8-1.9-fold, Fig. 4A), which is strongly affected by alterations in the mesophyll anatomy (Fig. 5A); the significant increase in the number of chloroplasts (1.5-fold) with a decrease in chloroplast size (Table 1) resulted in a 1.6-fold increase in the chloroplast surface area (*S*_c_, Table 1) for CO_2_ diffusion^5^. The concurrent increases in *S*_c_ and *g*_m_ in the *EcHB1*-overexpressed lines (Fig. 4C), together with the concurrent increases in *g*_m_ and *A* (Fig. 4C), supports the significant role of *S*_c_ for determining *g*_m_, and thus, photosynthesis. On the other hand, overexpression of *EcHB1* induced little alteration in the stomatal conductance, *g*_s_ (Fig. 4A). The stomatal density and size strongly affect stomatal conductance, *g*_s_^24^. Low stomatal density as well as large stomatal size reduces *g*_s_, because low stomatal density reduces the total stomatal pore area in leaves, while large stomatal size increases the diffusion path length^25^. The small change in stomatal conductance (*g*_s_) by the overexpression of *EcHB1* (Fig. 4A) may be due to the lack of alterations in both the stomatal density and size (Table 1). CO_2_ diffusion through the stomata and mesophyll imposes almost comparable limitations in photosynthesis in different tree groups including deciduous, evergreens, and conifers^7^. The present study for *Eucalyptus* trees showed that the mesophyll tended to impose lower limitations in the overexpression lines EcHB1-2 and EcHB1-10 compared to GUT5 (Fig. 4B). This result reflects the enhancement of *g*_m_ by the overexpression of *EcHB1* (Fig. 4A).

Overexpression of *EcHB1* improves the drought tolerance of *Eucalyptus* trees (Fig. 6). This improvement in drought tolerance may be related to the alteration in the transpiration of the whole tree. As a result of (1) an unchanged transpiration rate on a leaf-area basis, (2) a significant reduction in leaf area (0.5-fold), and (3) a small increase in the number of leaves (1.3-fold, data not shown), the estimated transpiration rate on a tree basis was reduced (0.6-fold) for the *EcHB1*-overexpressed lines (Table 2). This will reduce water loss from trees, and thus, may contribute to prevent the *EcHB1*-overexpressed lines from leaf-falling. Additionally, we observed alterations in the vessel anatomy in the stems of *EcHB1*-overexpressed lines, with a decrease in the radial diameter and a high cell wall thickness (data not shown). These changes will enhance stiffness of xylem cells but reduce the hydraulic conductivity in stems^26,27^, and as a result, will also reduce water loss from the trees during drought. Although the lower root biomass in the *EcHB1*-overexpressed lines (Fig. 1B,C) may cause less water uptake from the soil and thus potentially induce a sensitive response to drought^28^, the effect of reducing water loss (Table 2) may overcome the effect of less water uptake under the severe drought condition in the present study. The lower rate of leaf-falling during drought (Fig. 6A) may contribute the rapid recovery of the number of leaves after re-watering and thus improves the growth of the trees (Fig. 6C). Some morphological properties of the *EcHB1*-overexpressed lines on a tree-basis are similar to the *Eucalyptus* trees with high drought tolerance. *Eucalyptus* provenances from dry areas in Australia have a higher leaf per stem area ratio^29^, which supports our result of a larger number of leaves with a smaller stem diameter, i.e., a larger number of leaves per stem area, in the *EcHB1*-overexpressed line EcHB1-2 (Fig. 6A).

The HD-Zip I, HD-Zip II, and HD-Zip III transcription factor networks regulate plant growth responses to environmental conditions through suppression or promotion of cell multiplication, differentiation, and expansion through phytohormone-regulated networks^16,17^. Some *Arabidopsis* HD-Zip II genes are required for shade-induced hypocotyl elongation^30,31^, as well as controlling several auxin-regulated developmental processes including lateral organ polarity^32^. Overexpression of *EcHB1*, an HD-Zip II transcription factor, induced an increase in tree height, a decrease in tree diameter, and a decrease in root growth in *Eucalyptus* (Fig. 1B,C), which is in line with the results of a previous study^30^; in *Arabidopsis* seedlings, the increasing level of *Arabidopsis* HD-Zip II gene *AtHB2*, induces shifts from radial cell expansion to longitudinal cell expansion in the hypocotyl, and involves an inhibition in root growth. The increase in plant height by the overexpressing *EcHB1* under the control of CaMV 35 S promoter was also reported in tobacco plants^15^. These results suggest that the functions of *EcHB1* for plant growth are similar to those of *AtHB2*.

Alterations in leaf morphology by the overexpression of *EcHB1* (Fig. 2, Table 1) again suggests similar functions of *EcHB1* to those of some *Arabidopsis* HD-Zip II genes including *AtHB2*. The decreased leaf size in *EcHB1*-overexpressed *Eucalyptus* (Fig. 2) supports the results of previous studies which found that overexpression of *Arabidopsis* HD-Zip II genes, *AtHB2*, *AtHB4*, *HAT1*, *HAT2*, and *HAT3* decreased leaf expansion in *Arabidopsis*^30,33–36^. The reduction in leaf expansion may be partly due to the reduction in the width of epidermal cells in the leaf-width direction^30^ (Table 1). In the present study, we also found increases in the proliferation of leaf mesophyll cells, as well as increases in the number of chloroplasts in the leaf-thickness direction in the *Eucalyptus* plants overexpressing *EcHB1* (Table 1), which suggest additional functions of HD-Zip II genes in leaf development.

In conclusion, overexpression of *EcHB1*, an homeodomain leucine zipper (HD-Zip) II transcription factor, in the hybrid eucalypt GUT5 (*Eucalyptus grandis* and *Eucalyptus urophylla*) induced significant enhancements in leaf-area-based leaf photosynthesis. This improvement is due to both enhanced CO_2_ diffusion into chloroplasts and increased photosynthetic biochemical functions, which are affected by alterations in the mesophyll anatomy, e.g., an increased number of chloroplasts per unit leaf area. Overexpression of *EcHB1* improved drought tolerance, which is mainly affected by the reduction in leaf area with no changes in stomatal morphology. Although negative regulations of leaf size by *Arabidopsis* HD-Zip II genes have been already reported, the positive regulation of cell division and number of chloroplasts by an HD-Zip II gene are new findings, which gave us insights into the role of the HD-Zip II gene.

## Methods

### Plant material

A hybrid *Eucalyptus* tree GUT5 generated from *Eucalyptus grandis* and *Eucalyptus urophylla* was used as the host plant^14^. This GUT5 is a selected clone that has a high ability to regenerate from a transformed callus. GUT5 shows good growth performance in subtropical areas, especially in Brazil where commercial forest plantations of *Eucalyptus* species are widely performed. Transgenic *Eucalyptus* GUT5 lines with a CaMV 35S promoter fused to EcHB1 cDNA (accession No. AB458829) were generated in our previous study^15^. We analyzed control (GUT5) and two transgenic lines (EcHB1-2 and EcHB1-10), in which 3–16 plants were used for the growth and drought experiment and one - three individual were selected for the detailed analysis of leaves. To obtain multiple transgenic *Eucalyptus* plants with the same genetic background, the stems of *Eucalyptus* plants were transplanted to 1.5-liter plastic pots filled with soil and then grown in temperature-controlled greenhouses of the Forestry Research Institute of the Oji Holdings Corporation (Kameyama, Japan) and Kyoto Institute of Technology (Kyoto, Japan). The plants were watered daily and fertilized biweekly with liquid fertilizer Hyponex (Hyponex Japan, Osaka, Japan) at a concentration of 1/1000. In the glasshouse of the Forestry Research Institute, the daily minimum and maximum temperatures ranged from 15–25 °C and 25–36 °C, respectively. In the glasshouse of the Kyoto Institute of Technology, the temperature was kept at 25 °C. Prior to the detailed leaf analysis, phenotypes of the plants and the growth rate were measured at the Forestry Research Institute for 12-month-old trees. The growth rates in stem diameter and tree height were calculated as increments of the values per 12 months.

### Analysis of expression levels of *EcHB1* in the transgenic lines of *Eucalyptus* trees

For RT-PCR analysis, RNA samples were extracted using an RNeasy Plant Mini Kit (Qiagen, CA). RT-PCR was performed using a PrimeScript RT-PCR kit (Takara Bio, Shiga, Japan) and *EcHB1*-specific primers (HB1RTF and HB1RTR)^15^. As an internal control to assess cDNA yield between the RNA samples, glyceraldehyde-3-phosphate dehydrogenase (GAPDH)-specific primers (5′-TAGCCATTTCCAGAACCCTCG-3′ and 5′-CGGAGATGACAACCTTCTTAG-3′)^37^ were used. The procedure of northern blot analysis including RNA extraction was as described previously^15^.

### Measurement of leaf photosynthesis and stomatal and mesophyll conductance

Leaf photosynthesis was measured in the 4-year-old *Eucalyptus* trees, i.e., trees grown for four years since transformation at the Kyoto Institute of Technology, using the fully-expanded mature 5^th^ to 10^th^ leaves from the tops of the branches. The measurements were performed from August to October, in which four leaves from one each of individual trees of GUT5 and EcHB1-2 (*n* = 4), and four leaves from three individual trees of EcHB1-10 (*n* = 12) were used for the measurements. Photosynthetic parameters were measured using a photosynthesis system (Li-6400, Li-Cor, Lincoln, NE, USA), estimated from the photosynthesis (*A*)/intercellular CO_2_ concentration (*C*_i_) curve fit model (*A*/*C*_i_ curve fit model)^38,39^ and the light response curve model^40^, with the leaf temperature and vapor pressure deficit (VPD) being set at 25 °C and 1.5 kPa, respectively. *A*/*C*_i_ curves and light response curves were obtained at a photosynthetic photon flux density (PPFD) of 1500 µmol m^−2^ s^−1^ and at 400 µmol mol^−1^ of ambient CO_2_, respectively, as in a previous study^24^. The photosynthetic rate and stomatal conductance at light saturation (*A*_1500_, *g*_s-1500_) were obtained at a PPFD of 1500 µmol m^−2^ s^−1^.

The mesophyll conductance (*g*_m_) of the leaves of the *Eucalyptus* trees was measured using two methods: the isotope discrimination method for six leaves (*n* = 6) and the curve-fitting method for four to twelve leaves (*n* = 4–12), as described previously^8^. For the isotope discrimination method, measurements were performed from November to December, in which the equations provided by the previous study that take the ternary effect into account^41^ were used for the estimation of *g*_m_. The data for EcHB1-10 obtained by the isotope discrimination method were eliminated from the analysis, because unexpected senescence occurred at the time of the measurement in the leaves of EcHB1-10. The second estimate of *g*_m_ was obtained for all lines using the curve-fitting method^38^. The relative limitation of photosynthesis by stomatal conductance, mesophyll conductance, and biochemical capacity were estimated as in a previous study^6^.

### Leaf morphological and stomatal properties

For measurements of the leaf morphological traits, fully expanded 5^th^ to 10^th^ leaves from the top of the branches were obtained during the same period as the photosynthesis measurements were performed (from August to October). Leaf transverse sections were obtained from five leaves (*n* = 5) at the central part of the lamina, and fixed and embedded in Spurr’s resin. Mesophyll anatomy was analyzed in 800-nm-thick transverse sections of leaves, observed using a light microscope (BX51-33, OLYMPUS, Tokyo, Japan), and the cell wall thickness of mesophyll cells was observed in 80-nm-thick transverse sections of leaves with a transmission electron microscope (JEM-1220, JOEL, Tokyo, Japan), as described previously^42^. Leaf anatomical traits were measured from digitized images of leaf sections of the lines GUT5, EcHB1-2, and EcHB1-10, using software^43^ (Image J). The surface area of chloroplasts exposed to the intercellular air spaces per unit leaf area (*S*_c_) and mesophyll porosity were estimated as described previously using the images of light micrographs^10^. Briefly, *S*_c_ was estimated from the ratio of the perimeter length of chloroplasts/mesophyll cells exposed to the intercellular air spaces. Mesophyll fraction was calculated as 1 – mesophyll porosity. The number of mesophyll cells and chloroplasts, mesophyll thickness, mesophyll fraction, and *S*_c_ were measured from five images for each line (*n* = 5). Cell size (width and length) of the uppermost palisade mesophyll and the secondary layer of spongy mesophyll, the size of chloroplasts in palisade cells (width and thickness), and upper epidermal cell size (width and thickness) were measured in five cells of each image (*n* = 25). The thickness of the cell walls covered with chloroplasts was measured at two parts of the palisade cells in 10 images of sections of 80 nm thickness (*n* = 20) using a transmitting electron microscope (JEM-1220, JEOL Ltd., Tokyo, Japan). The chlorophyll content was estimated using a chlorophyll meter for six leaves (SPAD-502Plus, Konica-Minolta, Tokyo, Japan); the chlorophyll content was shown as SPAD values^44^. Leaf area was measured in four leaves using a scanner (Canoscan 9950 F, Canon, Tokyo, Japan) and image analysis software (Image J), and the leaves were dried at 60 °C for 48 h using an oven (MOV-112, SANYO Electric, Osaka, Japan) and weighed to assess the dry mass to calculate leaf dry mass per leaf area (LMA).

Stomatal density (SD) and stomatal size (length and width) were estimated in the lines GUT5 and EcHB1-10 using light micrographs of the replicas of leaf surface, as described in our previous paper^24^. The fully-expanded 5^th^ leaves from the top of the branches were obtained in July, and SD was measured in two leaves of the four individuals (*n* = 8), and stomatal sizes were measured in five closed stomata of two leaves from the three individuals (*n* = 30).

### Tree-basis photosynthesis and evaporation

The Leaf-area-basis photosynthesis rate and evaporation rate (*A*_1500_, *E*_1500_) measured at 1500 μmol m^−2^ s^−1^ of photosynthetic photon flux density (PPFD) and *S*_c_ were obtained for four leaves of the GUT5 and EcHB1-2 lines (*n* = 4), as described previously. Then, whole-leaf-basis *A*_1500_, *E*_1500_, and *S*_c_ were calculated using the averaged leaf-area-basis data multiplied by the leaf area of six leaves (*n* = 6). Tree-basis *A*_1500_, *E*_1500_, and *S*_c_ were calculated using the averaged whole-leaf-basis data multiplied by the number of leaves of the 8-month-old individual trees (*n* = 15–16).

### Drought tolerance experiment

Seven to nine *Eucalyptus* trees of the lines GUT5 and EcHB1-2 were generated from tree cuttings or calli, and grown in 7.0-liter pots for eight months in the glasshouse of the Forestry Research Institute of Oji Holdings Corporation, and irrigated with sufficient amounts of water daily before the drought experiment. To determine drought tolerance, drought and recovery experiments were successively performed. For the drought experiment, *Eucalyptus* trees were grown for 4 months under low-, middle-, and high-drought levels. The “low-drought level” was determined as the minimum irrigation level that no leaf shedding occurs, which was irrigation of 870 mL of water per each pot every other day. For the middle- and high-drought levels, irrigation of 870 mL of water was reduced every two days and every four days, respectively. Thereafter, irrigation with a sufficient amount of water was started again every two days (recovery experiment). Tree heights, diameters at the base of the stems, and number of leaves were measured monthly during the drought experiment, and then, tree height and shoot and stem fresh weight were measured after the recovery experiment. The duration of the drought and recovery experiment was determined as simulated dry and wet periods in the commercial plantation site for *Eucalyptus* in Brazil.

### Statistical analysis

Differences between the *Eucalyptus* transgenic lines (EcHB1-2 and EcHB1-10) and the control line GUT5 were tested using ANOVA with Dunnett’s post hoc test. The differences in the traits between GUT5 and the pooled data for the two transgenic lines EcHB1-2 and EcHB1-10 were tested using Welch’s *t*-test. Principal component analyses were performed to analyze 1) patterns of similarities in the photosynthetic traits and some anatomical traits and 2) patterns of similarities in the leaf anatomical traits. These statistical analyses were performed using R software (ver. 3.2.4)^45^.

## Supplementary information

Overexpressing the HD-Zip class II transcription factor EcHB1 from *Eucalyptus camaldulensis*increased the leaf photosynthesis and drought tolerance of *Eucalyptus*

## References

[1] Celso, F. Papermaking properties of Eucalyptus trees, woods, and pulp fibers. *Eucalyptus Online B*. *Newsl*. 1–110 (2009).

[2] Global Forest Watch. Tree Plantations. Available at, https://www.globalforestwatch.org/ (2018).

[3] Stape JL, Binkley D, Ryan MG (2004). Eucalyptus production and the supply, use and efficiency of use of water, light and nitrogen across a geographic gradient in Brazil. For. Ecol. Manage..

[4] Almeida AC, Soares JV, Landsberg JJ, Rezende GD (2007). Growth and water balance of Eucalyptus grandis hybrid plantations in Brazil during a rotation for pulp production. For. Ecol. Manage..

[5] Terashima I, Hanba YT, Tholen D, Niinemets Ü (2011). Leaf functional anatomy in relation to photosynthesis. Plant Physiol.

[6] Grassi G, Magnani F (2005). Stomatal, mesophyll conductance and biochemical limitations to photosynthesis as affected by drought and leaf ontogeny in ash and oak trees. Plant, Cell Environ..

[7] Flexas J (2012). Mesophyll diffusion conductance to CO_2_: An unappreciated central player in photosynthesis. Plant Sci..

[8] Tosens T (2016). The photosynthetic capacity in 35 ferns and fern allies: mesophyll CO_2_ diffusion as a key trait. New Phytol..

[9] Kogami H (2001). CO_2_ transfer conductance, leaf structure and carbon isotope composition of Polygonum cuspidatum leaves from low and high altitudes. Plant Cell Environ..

[10] Hanba YT, Kogami H, Terashima I (2002). The effect of growth irradiance on leaf anatomy and photosynthesis in Acer species differing in light adaptation. Plant Cell Environ..

[11] Peguero-Pina JJ (2016). Light acclimation of photosynthesis in two closely related firs (*Abies pinsapo* Boiss. and *Abies alba* Mill.): The role of leaf anatomy and mesophyll conductance to CO_2_. Tree Physiol..

[12] Flexas J (2016). Mesophyll conductance to CO_2_ and Rubisco as targets for improving intrinsic water use efficiency in C_3_ plants. Plant. Cell Environ..

[13] Xiong D, Douthe C, Flexas J (2018). Differential coordination of stomatal conductance, mesophyll conductance, and leaf hydraulic conductance in response to changing light across species. Plant Cell Environ..

[14] Tsuchihira A (2010). Effect of overexpression of radish plasma membrane aquaporins on water-use efficiency, photosynthesis and growth of Eucalyptus trees. Tree Physiol..

[15] Sonoda T (2009). Increasing fiber length and growth in transgenic tobacco plants overexpressing a gene encoding the Eucalyptus camaldulensis HD-Zip class II transcription factor. Plant Biotechnol..

[16] Harris JC, Hrmova M, Lopato S, Langridge P (2011). Modulation of plant growth by HD-Zip class I and II transcription factors in response to environmental stimuli. New Phytol..

[17] Turchi L, Baima S, Morelli G, Ruberti I (2015). Interplay of HD-Zip II and III transcription factors in auxin-regulated plant development. J. Exp. Bot..

[18] Girijashankar V (2011). Genetic transformation of eucalyptus. Physiol. Mol. Biol. Plants.

[19] Hu WJ (1999). Repression of lignin biosynthesis promotes cellulose accumulation and growth in transgenic trees. Nat. Biotechnol..

[20] Pilate G (2002). Field and pulping performances of transgenic trees with altered lignification. Nat. Biotechnol..

[21] Vanholme R, Morreel K, Ralph J, Boerjan W (2008). Lignin engineering. Curr. Opin. Plant Biol..

[22] Voelker SL, Lachenbruch B, Meinzer FC, Kitin P, Strauss SH (2011). Transgenic poplars with reduced lignin show impaired xylem conductivity, growth efficiency and survival. Plant, Cell Environ..

[23] Carriquí M (2015). Diffusional limitations explain the lower photosynthetic capacity of ferns as compared with angiosperms in a common garden study. Plant, Cell Environ..

[24] Kiyomizu T, Yamagishi S, Kume A, Hanba YT (2019). Contrasting photosynthetic responses to ambient air pollution between the urban shrub Rhododendron × pulchrum and urban tall tree Ginkgo biloba in Kyoto city: stomatal and leaf mesophyll morpho-anatomies are key traits. Trees.

[25] Franks PJ, Beerling DJ (2009). Maximum leaf conductance driven by CO_2_ effects on stomatal size and density over geologic time. Proc. Natl. Acad. Sci..

[26] Lachenbruch B, Mcculloh KA (2014). Traits, properties, and performance: How woody plants combine hydraulic and mechanical functions in a cell, tissue, or whole plant. New Phytol..

[27] Santini NS (2016). Xylem traits and water-use efficiency of woody species co-occurring in the Ti Tree Basin arid zone. Trees - Struct. Funct..

[28] Costa e Silva F (2004). Responses to water stress in two *Eucalyptus globulus* clones differing in drought tolerance. Tree Physiol..

[29] Li C, Berninger F, Koskela J, Sonninen E (2000). Drought responses of Eucalyptus microtheca provenances depend on seasonality of rainfall in their place of origin Chunyang. Aust. J. Plant Physiol..

[30] Steindler C (1999). Shade avoidance responses are mediated by the ATHB-2 HD-Zip protein, a negative regulator of gene expression. Development.

[31] Carabelli, M., Turchi, L., Ruzza, V., Morelli, G. & Ruberti, I. Homeodomain-Leucine zipper II family of transcription factors to the limelight: Central regulators of plant development. *Plant Signal*. *Behav*. **8** (2013). 10.4161/psb.25447 PMC4002598 23838958

[32] Turchi L (2013). Arabidopsis HD-Zip II transcription factors control apical embryo development and meristem function. Development.

[33] Sawa S (2002). The HAT2 gene, a member of the HD-Zip gene family, isolated as an auxin inducible gene by DNA microarray screening, affects auxin response in Arabidopsis. Plant J..

[34] Sorin C, Salla-Martret M, Bou-Torrent J, Roig-Villanova I, Martínez-García JF (2009). ATHB4, a regulator of shade avoidance, modulates hormone response in Arabidopsis seedlings. Plant J..

[35] Ciarbelli AR (2008). The Arabidopsis Homeodomain-leucine Zipper II gene family: Diversity and redundancy. Plant Mol. Biol..

[36] Bou-Torrent J (2012). ATHB4 and HAT3, two class II HD-ZIP transcription factors, control leaf development in Arabidopsis. Plant Signal. Behav..

[37] Fairbairn D (2000). Characterisation of two distinct HKT1-like potassium transporters from Eucalyptus camaldulensis. Plant Mol. Biol..

[38] Ethier GJ, Livingston NJ (2004). On the need to incorporate sensitivity to CO_2_ transfer conductance into the Farquhar-von Caemmerer-Berry leaf photosynthesis model. Plant, Cell Environ..

[39] Ethier GJ, Livingston NJ, Harrison DL, Black TA, Moran JA (2006). Low stomatal and internal conductance to CO_2_ versus Rubisco deactivation as determinants of the photosynthetic decline of ageing evergreen leaves. Plant, Cell Environ..

[40] Ögren E, Evans JR (1993). Photosynthetic light-response curves. Planta.

[41] Evans JR, Von Caemmerer S (2013). Temperature response of carbon isotope discrimination and mesophyll conductance in tobacco. Plant, Cell Environ..

[42] Nishida K, Kodama N, Yonemura S, Hanba YT (2015). Rapid response of leaf photosynthesis in two fern species *Pteridium aquilinum* and *Thelypteris dentata* to changes in CO2 measured by tunable diode laser absorption spectroscopy. J. Plant Res..

[43] Schneider CA, Rasband WS, Eliceiri KW (2012). NIH Image to ImageJ: 25 years of image analysis. Nature Methods.

[44] Markwell J, Osterman JC, Mitchell JL (1995). Calibration of the Minolta SPAD-502 leaf chlorophyll meter. Photosynth. Res..

[45] R_Core_Team. R: A language and environment for statistical computing (2016).

