# Supplementary material for: Overexpressing the HD-Zip class II transcription factor EcHB1 from Eucalyptus camaldulensis increased the leaf photosynthesis and drought tolerance of Eucalyptus
Source: Sci Rep. 2019 Oct 1;9:14121. doi: 10.1038/s41598-019-50610-5 (PMC6773882; doi:10.1038/s41598-019-50610-5)
Supplement: Supplementary file 1 — Overexpressing the HD-Zip class II transcription factor EcHB1 from Eucalyptus camaldulensisincreased the leaf photosynthesis and drought tolerance of Eucalyptus [file 41598_2019_50610_MOESM1_ESM.pdf]

Overexpressing the HD-Zip class II transcription factor EcHB1 from *Eucalyptus camaldulensis* increased the leaf photosynthesis and drought tolerance of *Eucalyptus*

Keisuke Sasaki, Yuuki Ida, Sakihito Kitajima, Tetsu Kawazu, Takashi Hibino, Yuko T. Hanba

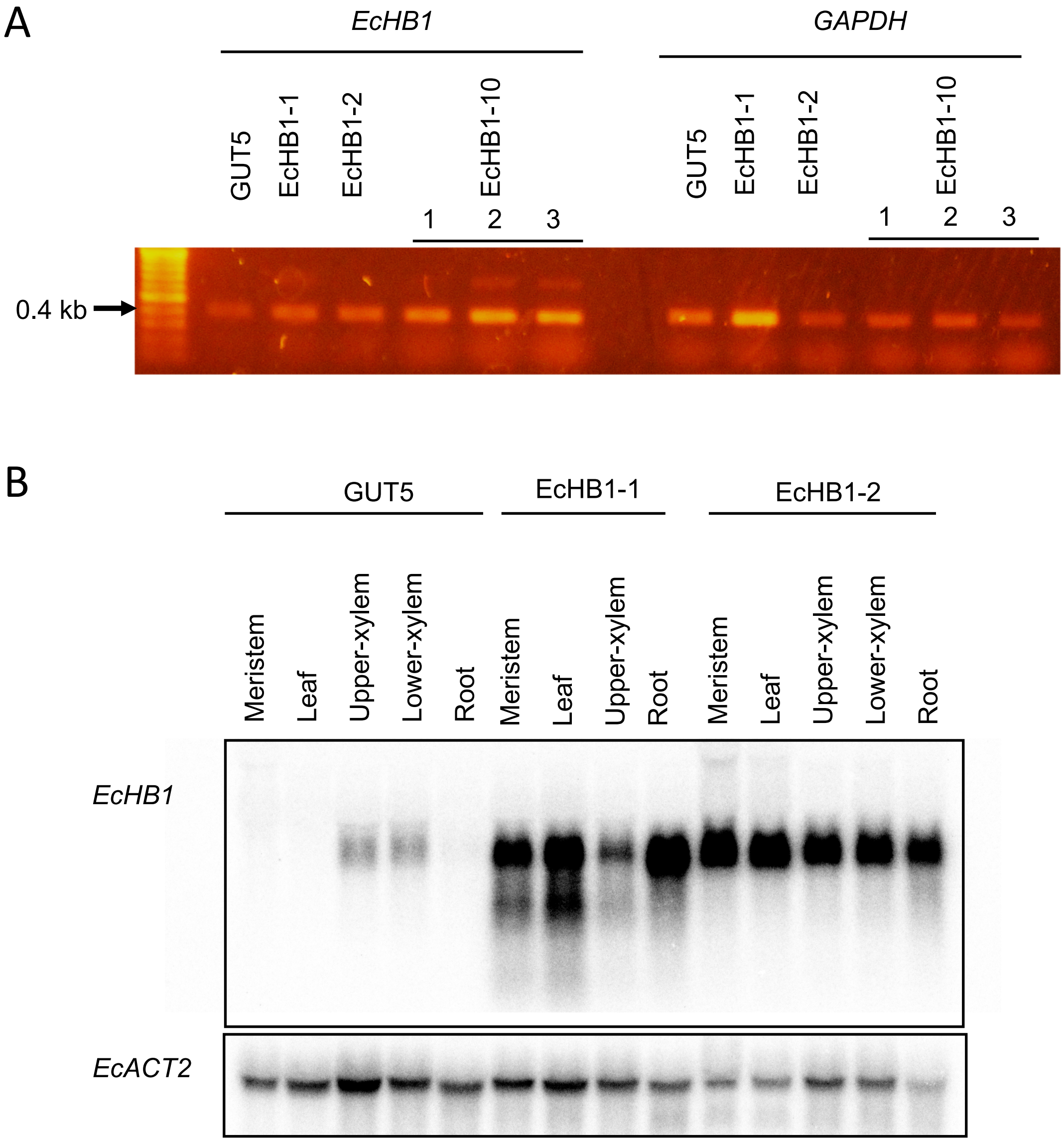

Fig. S1. (**A**) RT-PCR analysis for relative expression levels of *EcHB1* and the internal control GAPDH (0.4 kb) in the leaves of *Eucalyptus* trees for the control GUT5 and EcHB1-overexpressed lines EcHB1-1, EcHB1-2 and EcHB1-10. For the line EcHB1-10, three individual trees were used for the analysis. The total RNA fraction was prepared from 100 mg of leaf tissue obtained from a mature, fully-expanded leaf. (**B**) Northern blot analysis of the meristem, leaf, upper xylem, lower xylem, and root of the control GUT5 and EcHB1-overexpressed lines EcHB1-1 and EcHB1-2. Total RNA was extracted from plant tissues obtained from 12-month-old *Eucalyptus* trees. The blots for EcHB1 and EcACT2 (control) were obtained from the different part of the same gel.
